# Root morphogenic pathways in *Eucalyptus grandis* are modified by the activity of protein arginine methyltransferases

**DOI:** 10.1186/s12870-017-1010-x

**Published:** 2017-03-09

**Authors:** Krista L. Plett, Anita E. Raposo, Stephen Bullivant, Ian C. Anderson, Sabine C. Piller, Jonathan M. Plett

**Affiliations:** 1 Hawkesbury Institute for the Environment, Western Sydney University, Richmond, NSW 2753 Australia; 2 School of Science and Health, Western Sydney University, Penrith, NSW 2751 Australia

**Keywords:** *Eucalyptus grandi*s, Root hair initiation, Root architecture, Cytoskeleton

## Abstract

**Background:**

Methylation of proteins at arginine residues, catalysed by members of the protein arginine methyltransferase (PRMT) family, is crucial for the regulation of gene transcription and for protein function in eukaryotic organisms. Inhibition of the activity of PRMTs in annual model plants has demonstrated wide-ranging involvement of PRMTs in key plant developmental processes, however, PRMTs have not been characterised or studied in long-lived tree species.

**Results:**

Taking advantage of the recently available genome for *Eucalyptus grandis*, we demonstrate that most of the major plant PRMTs are conserved in *E. grandis* as compared to annual plants and that they are expressed in all major plant tissues. Proteomic and transcriptomic analysis in roots suggest that the PRMTs of *E. grandis* control a number of regulatory proteins and genes related to signalling during cellular/root growth and morphogenesis. We demonstrate here, using chemical inhibition of methylation and transgenic approaches, that plant type I PRMTs are necessary for normal root growth and branching in *E. grandis*. We further show that *EgPRMT1* has a key role in root hair initiation and elongation and is involved in the methylation of β-tubulin, a key protein in cytoskeleton formation.

**Conclusions:**

Together, our data demonstrate that PRMTs encoded by *E. grandis* methylate a number of key proteins and alter the transcription of a variety of genes involved in developmental processes. Appropriate levels of expression of type I PRMTs are necessary for the proper growth and development of *E. grandis* roots*.*

**Electronic supplementary material:**

The online version of this article (doi:10.1186/s12870-017-1010-x) contains supplementary material, which is available to authorized users.

## Background

Post-translational modifications of proteins, including phosphorylation, acetylation, methylation or ubiquitination, have significant effects on both the structure and the function of proteins. Methylation of proteins typically occurs at arginine or lysine residues, catalysed by protein arginine methyltransferases (PRMTs) or protein lysine methyltransferases (PKMTs), respectively. Both families of enzymes use S-adenosyl-L-methionine (SAM) as a methyl donor to add one or more methyl groups to amines within the protein residue. Such modifications alter the end use of the protein [1, 2]. There are 11 identified members of the PRMT family, including the plant specific PRMT10 [3], which are classified into four different types based on the site(s) at which the arginine residue is methylated [4]. Type I PRMTs, produce monomethyl arginines (MMA) as an intermediate and asymmetric dimethyl arginines (aDMA). Type II PRMTs produce MMAs as an intermediate and symmetric dimethyl arginines (sDMA), while Type III PRMTs only make MMAs [5]. Type IV PRMTs methylate the secondary amine on the arginine and have only been described in yeast [4].

PRMTs are well conserved throughout all eukaryotic cells and their downstream effects include altered transcription, RNA processing, transport and translation, signal transduction, DNA repair, chromatin structure and cellular differentiation [3, 4]. Defects in PRMT expression have also been implicated in serious mammalian diseases [5, 6]. Protein targets of PRMTs are often histones resulting in altered gene transcription [7], although many non-histone methylated proteins have been identified [2, 8]. These non-histone proteins are often involved in RNA binding or transcription [9, 10], but also include cytoplasmic proteins involved in various cellular developmental processes [8]. While these proteins have been well characterised in mammalian- and yeast-based systems, our understanding of PRMT activity in regulating plant development and signalling is still developing [3]. In plants, Type I PRMTs have been implicated in the alteration of transcription through the methylation of histones (*PRMT1* homologues; [11, 12]). These histone modifications have been found to affect flowering time, among other processes (*PRMT4* or *PRMT10* homologues; [13–15]). PRMTs are also involved in RNA processing and ribosomal biogenesis in *Arabidopsis* (*PRMT3* homologue; [16]). *AtPRMT5*, a Type II PRMT, has been found to affect pre-mRNA splicing [17, 18], flowering time [18, 19], salt stress tolerance [20], primary root length [21], root stem cell maintenance during DNA damage [22] and circadian rhythms [23].

The majority of PRMT research in plants has involved the annual plant *Arabidopsis thaliana*. While many genomic resources exist in *Arabidopsis*, we were interested in annotating the PRMTs in a perennial tree model and characterizing their expression and effects on root formation. With a recently sequenced genome [24] and the ability to manipulate it genetically*,* the economically important tree model *E. grandis* presents a useful system to study the effects of PRMTs in a longer-lived plant species. We found that the *E. grandis* genome encodes a set of seven PRMTs, giving it one of the smallest complements of PRMTs in a sequenced plant species, and that these PRMTs are expressed in all major plant tissues. Using both chemical inhibition and transgenic modification of PRMT activity in *E. grandis*, we explored the role of Type I PRMTs in root growth and development. We found that transgenic repression of *EgPRMT1, 3*, *4* or *10* homologues result in a similar phenotype: interruption of the normal growth and branching of plant roots. Additionally, over-expression of *EgPRMT1* causes abnormal root hair extension. We demonstrate that plant roots over-expressing *EgPRMT1* have increased methylation of β-tubulin, which has been proposed to affect microtubule stability in neurons [25] and is a likely contributor to the root hair phenotype. Transcriptomic and proteomic data show that PRMTs act as key regulators of gene networks and pathways involved in the control of root growth and morphogenesis. Given the essential role of the root system, the study of PRMTs will be an important avenue of research to understand not only root patterning but also other aspects of plant health and nutrition.

## Results

### *E. grandis* encodes *PRMT*-like genes that exhibit similar expression patterns in major plant tissues

**Fig. 1:**
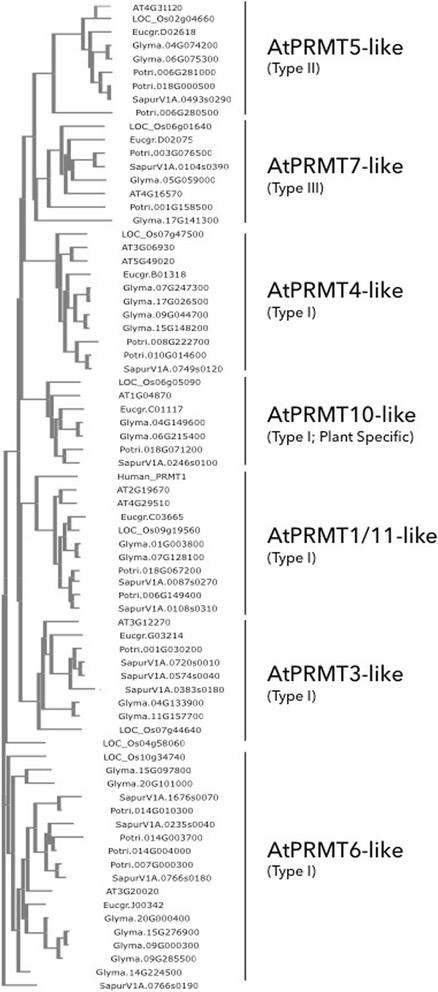
Genes homologous to *Arabidopsis thaliana PRMT*s have been conserved throughout the evolution of different plant lineages. Homologues of PRMTs encoded by *A. thaliana* (AT) are found in a range of plant genomes including *Eucalyptus grandis* (Eucgr); *Populus trichocarpa* (Potri); *Oryza sativa* (LOC_Os), *Glycine Max* (Glyma), and *Salix purpurea* (Sapur)

**Fig. 2:**
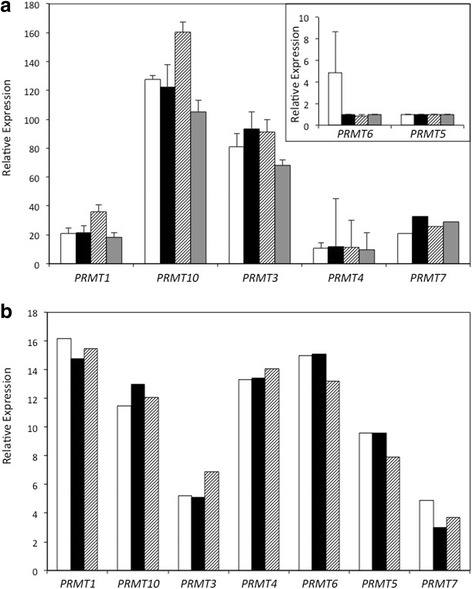
Relative expression of *PRMT* genes in *E. grandis* tissues. **a** Relative expression of *PRMT* genes in *E. grandis* seedling tissues; shoot apex (*white*), stem (*black*), leaf (hatched), root (*dark grey*) as determined by quantitative PCR compared to two housekeeping genes (Eucgr.K02046 and Eucgr.C00350; ± SE). **b** Relative expression of *PRMT* genes in mature *E. grandis* tissues; shoot apex (*white*), stem (*black*), leaf (hatched). Relative expression was based on normalized expression from RNA sequencing (FPKM) sourced from Mizrachi et al. [61].

By searching for the conserved functional domains common to arginine methyltransferases, seven *PRMT*-like genes were found within the *E. grandis* genome (v. 2.0; [24]). These include Eucgr.C03665.1 (*AtPRMT1* homologue), Eucgr.G03214.1 (*AtPRMT3* homologue), Eucgr.B01318.1 (*AtPRMT4* homologue), Eucgr.D02618.1 (*AtPRMT5* homologue), Eucgr.J00342.2 (*AtPRMT6* homologue), Eucgr.D02075.1 (*AtPRMT7* homologue) and Eucgr.C01117.1 (*AtPRMT10* homolog). These *E. grandis* genes have been named here according to their similarity to *A. thaliana PRMT*s and will be referred to as *EgPRMT*# throughout this paper. *E. grandis* had the smallest number of *PRMT*-like genes as compared to five other model plant species: *Arabidopsis thaliana* (9 PRMT genes; TAIR10; [26]), *Oryza sativa* (8 PRMT genes; v7.0; [27]), *Glycine Max* (21 PRMT genes; Wm82.a2.v1; [28]), *Populus trichocarpa* (14 PRMT genes; v3.0; [29]), and *Salix purpurea* (13 PRMT genes; v1.0; Fig. 1). Leaf, stem, apical and root tissues were collected from 10 week-old *E. grandis* seedlings and the relative expression of seven of the *PRMT* genes in each tissue type was determined by qPCR (Fig. 2a)*.* All *E. grandis* PRMT genes tested were expressed in each tissue type with *EgPRMT10* being the most highly expressed followed by *EgPRMT3*. Two genes (*EgPRMT6* and *EgPRMT5*) exhibited the lowest expression in all tissues analyzed (Fig. 2a, inset). In mature plants, *EgPRMT1* was the most highly expressed PRMT gene with *EgPRMT3* and *EgPRMT7* showing the lowest expression levels (Fig. 2b). In both seedlings and mature plants, each of the PRMT genes showed nearly identical expression levels between the different tissues.

### *E. grandis* plant tissues contain a diverse set of asymmetrically dimethylated proteins

Immuno-precipitation (IP) of asymmetrically dimethylated proteins was performed using the ASYM24 antibody (Merck-Millipore) and identified with mass spectrometry (Table 1). The majority of methylated proteins identified via mass spectrometry have roles in cellular respiration, although a number of proteins detected have been associated with the control of root growth and branching (e.g. heat shock protein 70 and 14-3-3-like proteins; [30–32]) and in the control of defence (endochitinases; [33]). It should be noted that not all proteins detected in the elutions of the immuno-precipitation column had a methylated arginine residue confirmed with mass spectrometry, but would have to be either asymmetrically di-methylated, or associate with an asymmetrically di-methylated protein, based on the affinity purification with the ASYM24 antibody. Proteins identified as methylated from the whole cell lysates could have been created by either type I or type II PRMTs as mass spectrometry data do not allow a differentiation between symmetric and asymmetric methylation.

**Table 1:** List of methylated proteins found in *E. grandis* root or leaf tissues

	Protein	Gene No.	RPKM/FPKM	Score	Methylated Peptide Sequences Identified by Mass Spectrometry	Predicted MW (kDa)
Root Proteins	14-3-3-like protein ^A^	Eucgr.B00034.1	254	431^1^	^165^AAQDIANSELAPTHPIR	29
	5-methyltetrahydropteroyltriglutamate-homocysteine methyltransferase ^A^	Eucgr.J00612.1	30	802^1^, 47.16^2^	^33^ISTGTGTGTGT**R**R	91
	Aspartic proteinase nepenthesin-like protein ^B^	Eucgr.G03227.1	147	43.97^2^		47
	ATP synthase subunit beta, mitochondrial ^A,B^	Eucgr.G02224.1	102	668^1^	^163^VLNTGSPITVPVGR	60
	Endochitinase A2 ^A, B^	Eucgr.I01495.1	37	229^1^, 58.25^2^	^107^SFPAFGTTGDAAT**R**	33
	Glyceraldehyde-3-phosphate dehydrogenase ^A,B^	Eucgr.B00144.1	73	620^1^, 62.03^2^	^274^GILGYTEEDVVSTDFVGDSR	36
	Heat shock 70 kDa protein ^A^	Eucgr.J00023.1	210	480^1^, 70.88^2^	^40^TTPSYVGFTDSE**R** ^60^NQVAMNPVNTVFDAKR	59
	Heat shock 80 kDa protein ^A^	Eucgr.F03673.1	555	195^1^	^162^DTSGEVLGR	80
	Malic Enzyme ^A^	Eucgr.C01003.1	354	224^1^	^568^YAESCMYSPVYR	64
	Nucleoside diphosphate kinase 1^A^	Eucgr.D01220.1	270	387^1^	^112^NVIHGSDSVESARK	16
	Oxygen evolving enhancer protein 1^A^	Eucgr.I01025.1	31	38.53^2^	^17^VGRISSSQLR	35
Leaf Proteins	ATP synthase delta chain, chloroplastic ^B^	Eucgr.F04149.1	39	215^1^		24
	ATP synthase gamma chain, chloroplastic ^B^	Eucgr.E04053.1	20	104^1^, 27.46^2^		40
	ATP synthase subunit beta, chloroplastic ^A,B^	Eucgr.H02991.1	9	2526^1^, 80.18^2^	^1^MRINPTTSGPGVSTLEK	53
	Basic endochitinase A ^B^	Eucgr.I02271.1	3,414	72^1^		29
	Basic endochitinase C ^B^	Eucgr.I02246.1	282	31.8^2^		16
	Carbonic anhydrase, chloroplastic ^B^	Eucgr.I01790.1	97	116^1^, 66.31^2^		41
	Chlorophyll a-b binding protein ^A^	Eucgr.D00322.1	29	347^1^	^197^WAMLGALGCVFPELLA**R**	38
	Cytochrome B6 ^A^	Eucgr.E04205.1	10	258^1^	^136^IVTGVPEAIPVGSPVVELLRIGSASVGQSTLTR	24
	Fructose-bisphosphate aldolase ^A^	Eucgr.G01726.1	53	851^1^, 32.85^2^	^82^LASIGLENTEANRQAYR	42
	Glutamine synthetase ^B^	Eucgr.B01163.1	8	113^1^		48
	Mediator of RNA Polymerase II Transcription Subunit	Eucgr.J00025.1	1,039	687^1^	^123^VEIIANDQGNRTTPSYVAFTDTER	81
	Malate dehydrogenase, glyoxysomal^B^	Eucgr.H02358.1	53	251^1^		34
	Oxygen-evolving enhancer protein 3–2, chloroplastic ^B^	Eucgr.D00854.1	33	313^1^, 73.74^2^		24
	Phosphoglycerate kinase ^A,B^	Eucgr.F01476.1	22	564^1^, 63.64^2^	^173^ADDCIGPEVEKLVASLPEGGVLLLENVR	50
	Photosystem I reaction center subunit IV B, chloroplastic ^B^	Eucgr.B02162.1	67	111^1^		15
	Ribulose bisphosphate carboxylase large chain ^A^	Eucgr.C03525.1	49	1800^1^	^165^YG**R**PLLGCTIKPK ^195^GGLDFTKDDENVNSQPFMR ^340^DITLGFVDLVRDDFIEKDR ^436^DLA**R**EGXDII**R**	52
	Ribulose bisphosphate carboxylase small chain ^A,B^	Eucgr.B03013.1	23	410^1^	^155^I**R**IIGFDNKR	20
	Ribulose bisphosphate carboxylase/oxygenase activase ^A^	Eucgr.J01234.1	42	72.19^2^	^274^EENP**R**VPIIVTGNDFSTLYAPLI**R** ^311^ED**R**IGVCMGIFR ^351^A**R**VYDDEVRK	51
	Ribulose-phosphate 3-epimerase, chloroplastic ^B^	Eucgr.B00532.1	41	27.54^2^		29
	Thylakoid lumenal 29 kDa, chloroplastic ^A^	Eucgr.F00373.1	4	762^1^, 51.08^2^	^264^DKFSAIGFGP**R**QTATETLLAADPDVSPWVQK	37

List of proteins identified from whole cell lysates (A) or ASYM24 IP column elutions (B). Score determined by either Mascot (1) or Peaks (−10logP) (2). Methylated arginines within sequences indicated in bold (dimethylated) or underlined (monomethylated). Root expression data (RPKM) was taken from Plett et al. [62] and foliar expression data (FPKM) were obtained from https://phytozome.jgi.doe.gov/pz/portal.html#!info?alias=Org_Egrandis (accessed November 2016)

### Chemical inhibition of PRMT activity within roots alters root morphology

**Fig. 3:**
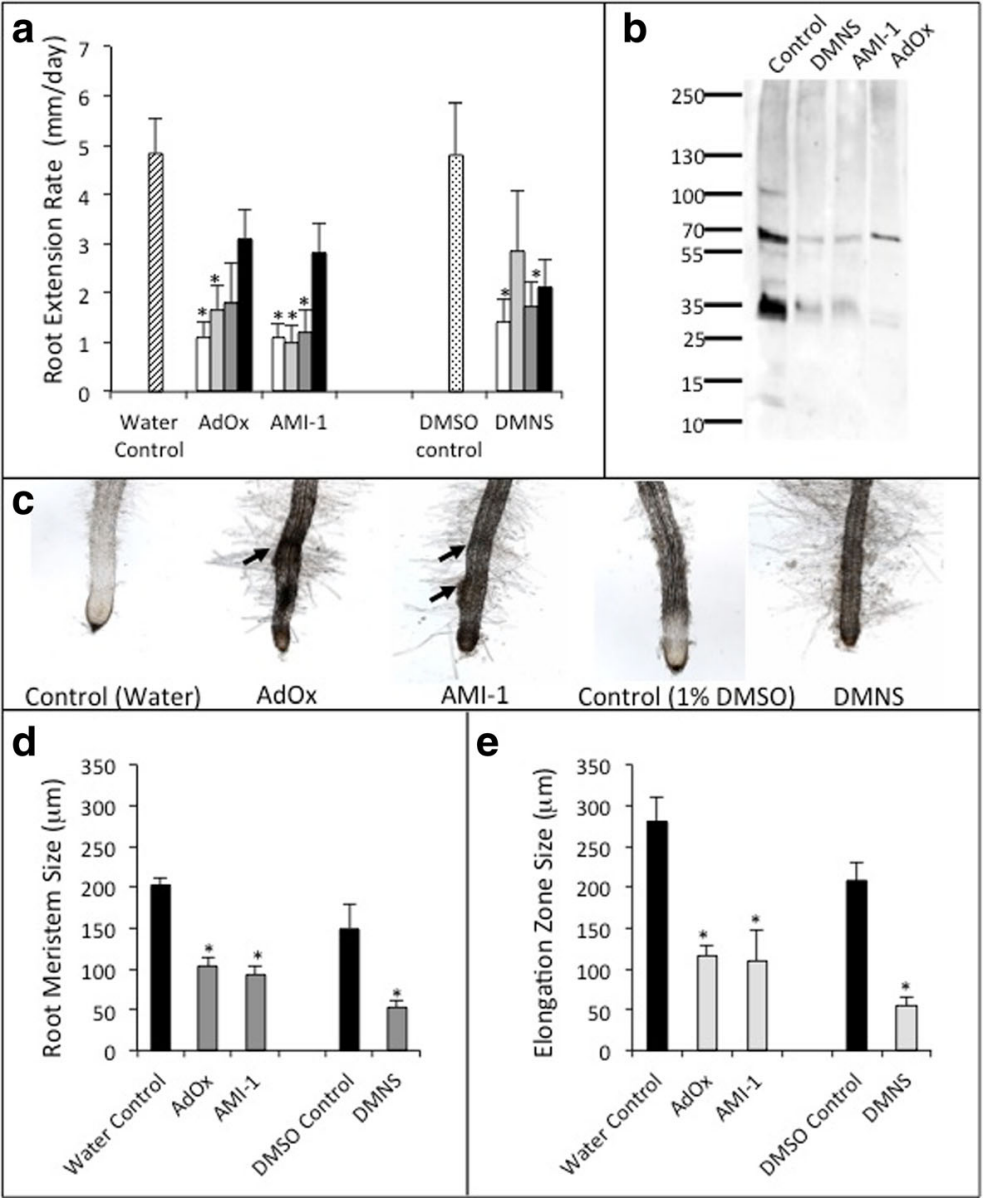
Effect of PRMT methylation inhibitor treatment on root morphology. **a** Rate of root growth of roots after 24 h of treatment with methylation inhibitors AdOx (5 μM *white bars*, 0.5 μM *light grey bars*, 50 nM *dark grey bars*, 5 nM *black bars*), AMI-1 (50 μM *white bars*, 5 μM *light grey bars*, 0.5 μM *dark grey bars*, 50 nM *black bars*) and 2,3-dimethoxynitrostyrene (DMNS; 30 μM *white bars*, 3 μM *light grey bars*, 0.3 μM *dark grey bars*, 30 nM *black bars* in 1% DMSO) or controls (water hatched bar or 1% DMSO cross hatched bar). **b** ASYM24 probed Western blot of total protein extracted from control or PRMT inhibitor treated roots. **c** Representative photos showing the morphology of roots treated with methylation inhibitors or controls. *Arrows* indicate abnormal root bulging observed in AdOx and AMI-1 treated roots. **d** Size of the root meristem after treatment with the highest concentration of PRMT inhibitor (*dark grey bars*) as compared to control treated roots (*black bars*). **e** Root elongation zone size after treatment with the highest concentration of PRMT inhibitor (*light grey bars*) as compared to control treated roots (*black bars*). ± SE; * = significant difference from corresponding control treatment (*p* < 0.05)

To determine if PRMTs play a role in root development, actively growing *E. grandis* roots were treated daily with one of three PRMT chemical inhibitors (adenosine dialdehyde [AdOx], arginine methyltransferase inhibitor 1 [AMI-1], 2,3-dimethoxynitrostyrene [DMNS]) over a two-week time period. All three inhibitors resulted in a significant reduction in root growth over the first 24 h (Fig. 3a) and resulted in a reduction of detectable methylated proteins as determined by Western blot using the anti-ASYM24 antibody (Fig. 3b). Roots treated with the general methylation inhibitor AdOx and with the PRMT general inhibitor AMI-1 exhibited bulging and irregular surface morphology (Fig. 3c). Roots treated with any of the three inhibitors exhibited a dense grouping of long root hairs just behind the elongation zone. Microscopic analysis of the root tips following inhibitor treatment showed a significant shortening of both the size of the root meristem and the length of the root cell elongation zone (Fig. 3d,e) with a rounder root tip and a smaller root cap.

### DMNS treated root tips have altered transcript abundance of genes related to root development

**Fig. 4:**
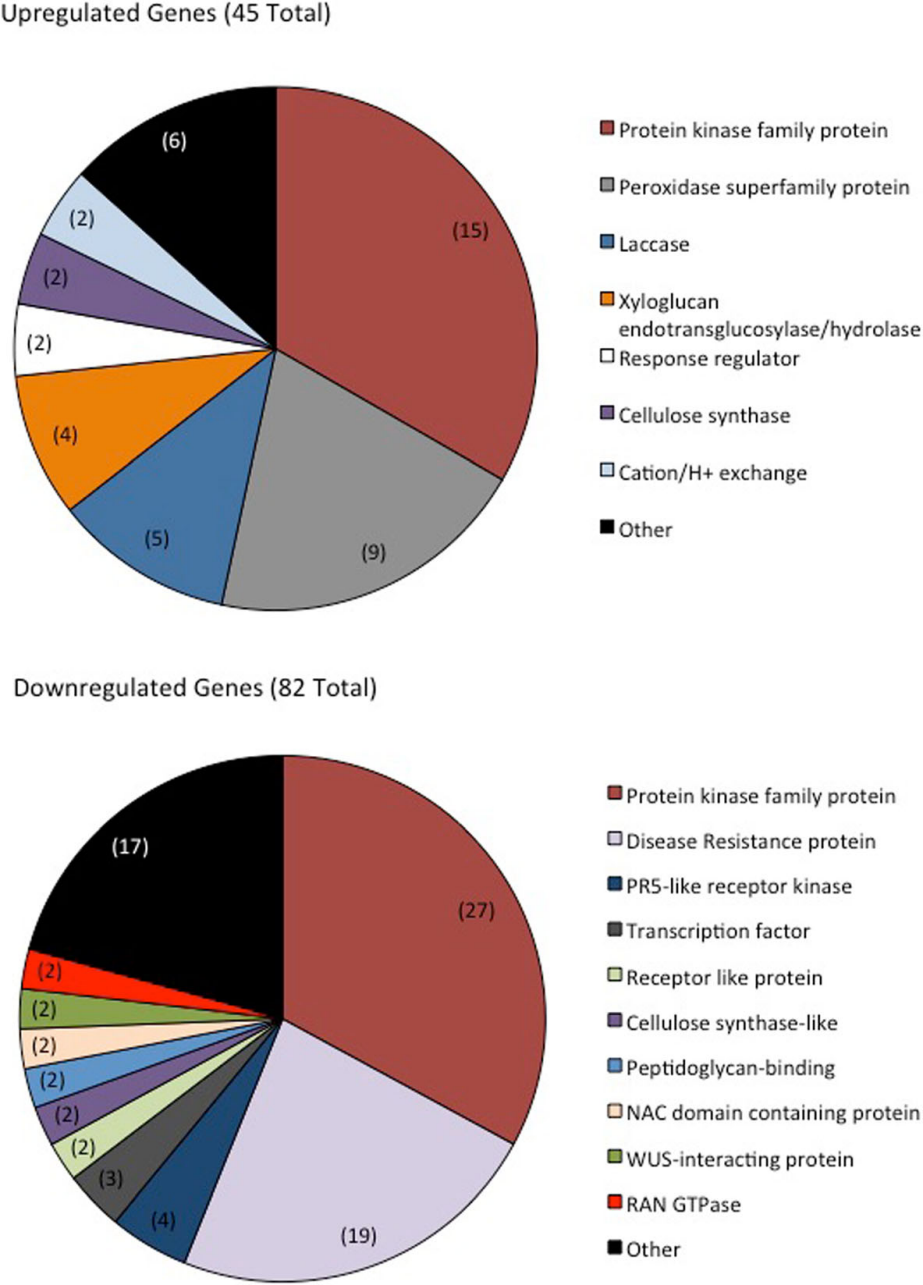
Classes of differentially-regulated genes pertaining to root development in DMNS treated roots. Representation of significantly (*p* < 0.05) regulated genes in 30 μM DMNS (in 1% DMSO) treated roots (as compared to control 1% DMSO treated roots) whose PFAM classifications are significantly over-represented and pertain to root development. Numbers in brackets represent number of genes in each category

RNA sequencing of DMNS treated roots found 873 differentially regulated genes as compared to the 1% DMSO treated controls (Additional file 1: Table S1). Of the differentially regulated genes, 554 were down-regulated (63.5%). PFAM enrichment analysis of the up- and down-regulated genes’ PFAM annotations found a significant enrichment (*p* < 0.001) for domains associated with root development (Additional file 2: Table S2). The corresponding genes made up 15.0% of all down-regulated genes and 14.1% of all up-regulated genes, with many having specified roles in cell wall organization, meristem development, auxin polar response, root hair elongation or multi-dimensional cellular growth. Of these genes, the largest group represented are kinases, making up about 33% of differentially regulated genes (Fig. 4; Additional file 2: Table S2).

### Transgenic roots differentially expressing PRMT genes show altered growth and lateral root formation

**Fig. 5:**
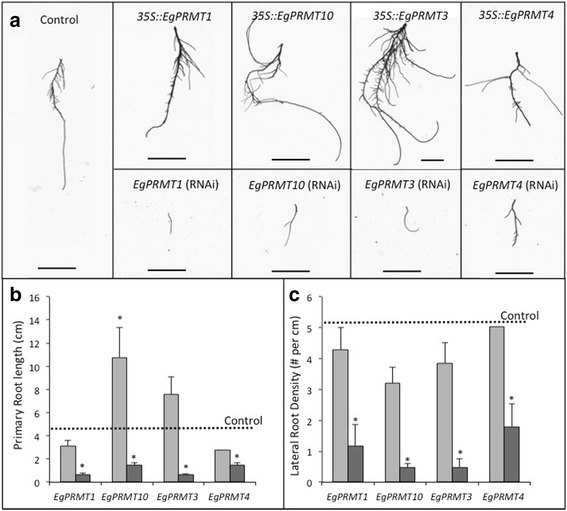
Effect of differential gene regulation of *EgPRMT*s on root growth and lateral branching. **a** Appearance of typical control or transformed roots either overexpressing (*35S::*) or underexpressing (RNAi) PRMT homologues. Scale bar = 1 cm; **b** Length of primary roots differentially expressing different PRMT genes (*35S:: −* *light grey*; RNAi – *dark grey*) averaged over all independent transformants generated. *Dashed line* represents average length of control primary roots. **c** Frequency of lateral root formation averaged over all independent transformants (*35S:: − light grey*; RNAi – *dark grey*). *Dashed line* represents average number of lateral roots per cm on control roots. ± SE; * = Significant difference from control (*p* < 0.05)

**Fig. 6:**
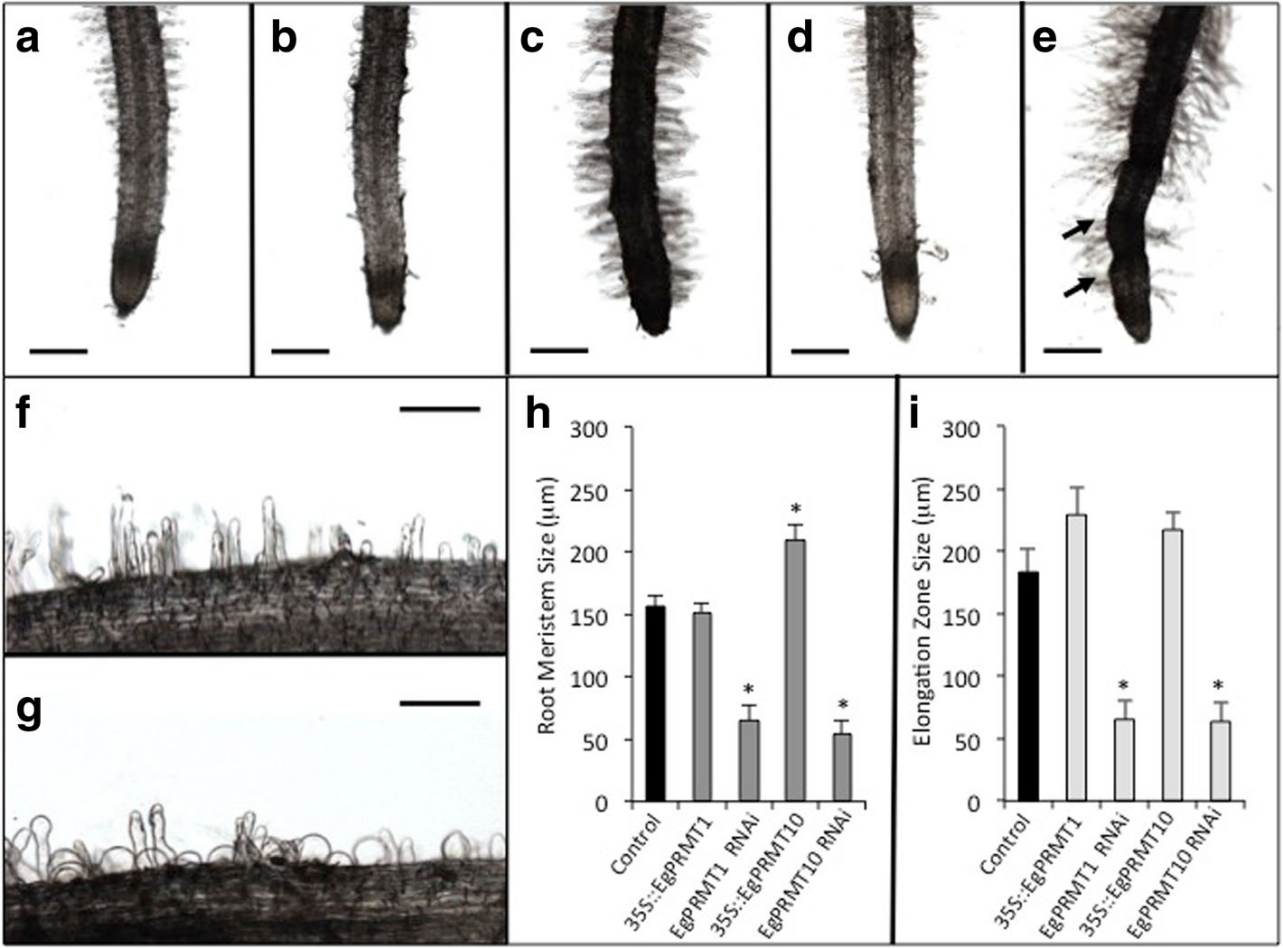
Effect of differential gene regulation of *EgPRMT*s on primary root and root hair morphology. **a** Control root; **b** *35S::EgPRMT1* root; **c** *EgPRMT1* RNAi root; **d** *35S::EgPRMT10* root; **e** *EgPRMT10* RNAi root. *Arrows* indicate abnormal bulging of roots. Expanded detail of control (**f**) and *35S::EgPRMT1* (**g**) root hairs. **h** Average change in length of meristem in control (*black*) and transgenic (*dark grey*) root tips. **i** Average change in length of elongation zone in control (*black*) and transgenic (*light grey*) root tips, ± SE. * = Significant difference from control (*p* < 0.05). Scale bars = 200 μm (**a**-**e**) or 100 μm (**f**-**g**)

Using an *Agrobacterium rhizogenes* mediated transformation, transgenic *E. grandis* roots were produced with either an overexpressing (*35S::*) or RNAi silenced (RNAi) version of the following Type I *PRMT* genes: *EgPRMT1*, *EgPRMT3*, *EgPRMT4* and *EgPRMT10* (Fig. 5; Additional file 3: Figure S2). Altered expression of the transgene in roots was confirmed by qPCR. Roots with elevated expression of *PRMT* genes showed unaltered (*35S::EgPRMT1, 3* and *4*) or significantly increased (*35S::EgPRMT10*) primary root length as compared to controls (Fig. 5b; Additional file 3: Figure S2). As seen in roots treated to chemically inhibit PRMT function, PRMT RNAi silenced roots were all significantly shorter than control roots. We also found that RNAi silenced roots showed a significant reduction in lateral rooting per unit length. Transgenic lines over-expressing the selected *PRMT* genes exhibited normal lateral root formation (Fig. 5c). Microscopic analysis of *EgPRMT1* and *EgPRMT10* transgenic root tips revealed that rounded root tips, as previously observed in chemically treated roots, were only observed in *EgPRMT1* RNAi lines (Fig. 6). We also observed alterations in root hair morphology and density (Fig. 6a–g). *35S::EgPRMT1* roots had predominantly short, rounded, swollen root hairs (Fig. 6g) while *35S::EgPRMT10* had normal root hairs with only occasional rounded root hairs. The *EgPRMT1* and *EgPRMT10* RNAi root hairs exhibited normal morphology, but appeared denser than controls (Fig. 6c,e). These roots also had increased pigmentation and, in the case of the *EgPRMT10* RNAi lines, displayed a bulbous, irregular root shape. As with the inhibitor treated roots, the meristem and expansion zones of the root tips in RNAi lines were significantly shorter than in controls (Fig. 6h, i).

### Transgenic roots expressing *35S::EgPRMT1* show increased β-tubulin methylation

**Fig. 7:**
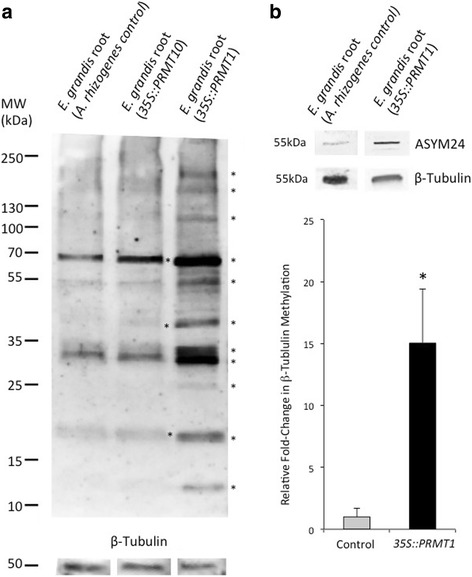
Western blot of un-treated *E. grandis* roots and roots transgenically mis-expressing *EgPRMT1* and *EgPRMT10.* **a** Western blots of whole cell lysates from *E. grandis* roots transformed with *A. rhizogenes* strain K599 (control) or roots over-expressing *EgPRMT1* or *EgPRMT10.* Blots were probed with anti-ASYM24. Equal amounts of total protein were migrated in each sample and *β-*tubulin was used to confirm normalization of total protein input. * = bands showing increased intensity from controls in multiple experiments. **b** Western blot of protein from *E. grandis* roots transformed with *A. rhizogenes* strain K599 (control) or *35S::EgPRMT1* immuno-precipitated with the anti-β-tubulin antibody. Blots were probed with anti-ASYM24 or anti-β-tubulin. Graph shows relative amount of methylated β-tubulin compared to total β-tubulin based on Western blot band intensity. * = significant increase in band intensity (*p* < 0.05)

Total protein was extracted from control and transgenic *35S::EgPRMT1* or *10* expressing roots. Protein extraction from RNAi lines was consistently unsuccessful, likely due to their small size and elevated pigmentation. Asymmetrically di-methylated proteins in *35S::* lines were visualized using Western blots with the ASYM24 antibody (Fig. 7a). When we analysed the Western blots of protein extracted from the transgenic roots, we identified several bands that were more intense in the *35S::EgPRMT1* Western blot as compared to controls. Our results, when comparing multiple extractions, indicated an increase in protein methylation of proteins with molecular weight of ~240, 190, 120, 70, 55, 30, 17 and 12 kDa (Fig. 7a, denoted by asterisks). We also observed the appearance of new methylated proteins at ~24 kDa and several between 35 and 70 kDa. The *35S::EgPRMT10* Western blot showed fewer proteins with increased arginine methylation compared to the *35S::EgPRMT1* blot but some were of similar molecular weight (~70, 40 and 17 kDa). Using these transgenic roots, we were able to identify an additional set of methylated proteins (Table 2). These include another heat shock protein (Hsp90) and α- and β-tubulin, proteins involved in root hair and cellular growth [34].

**Table 2:** List of methylated proteins found in *E. grandis* root tissues with elevated expression of *EgPRMT1* or *EgPRMT10*

	Protein	Gene No.	Score	Methylated Peptide Sequences Identified by Mass Spectrometry	Predicted MW (kDa)
*35S::PRMT1*	Endoplasmin/Heat Shock protein 90 kDa	Eucgr.H00972.1	611^1^, 162.29^2^	^531^ LGIIEDASNR	93
	RuBisCO large subunit- binding protein alpha	Eucgr.B02532.1	218^1^, 89.94^2^	^424^ VGAATESELEDR	69
	Alpha tubulin	Eucgr.B03604.1	85^1^, 92.43^2^	^65^ AIFLDLEPTVVDEVR	49
	Glyceraldehyde-3-phosphate dehydrogenase	Eucgr.H04673.1	499^1^, 164.68^2^	^66^ SISISISISIRPVR ^346^ RLEKAATYDEIK	36
	Probable aldo-keto reductase	Eucgr.H04367.1	79.32^2^	^249^ N**R**VVYAR	38
	Armadillo/beta catenin repeat protein	Eucgr.B00340.1	21.52^2^	^377^ IKLVKLNAVSALLGMLKSR	58
	40S Ribosomal Protein	Eucgr.A02371.1	117^1^	^194^ VLQFAGIDDVFTSSR	29
	Transaldolase family protein	Eucgr.G01978.1	277^1^	^78^ RTTLHDLYE**R**	47
	Probable Aldo-keto reductase	Eucgr.H04365.1	106^2^	^106^ GTPEYA**R**	38
	ATP synthase subunit beta	Eucgr.G02224.1	122^1^	^335^ DAEGQDVLLFIDNIF**R**	60
	Beta tubulin 5	Eucgr.D01847.1	481^1^	^155^ IREEYPDR	50
	Elongation factor 1-alpha	Eucgr.B02473.1	179^1^	^244^ IGGIGTVPVG**R**	49
	Phosphoenolpyruvate carboxylase	Eucgr.A01915.1	139.7^2^	^446^ **R**LDIR	110
	Heat shock protein 80 kDa	Eucgr.J00025.1	1341^1^, 211.26^2^	^131^ NRTTPSYVAFTDTER ^558^ FELSGIPPAPR	80
	Sucrose Synthase	Eucgr.H03515.1	536^1^, 133.9^2^	^772^ HVSNLDR	92
*35S::PRMT10*	Endochitinase A2	Eucgr.I01495.1	213^1^, 127.56^2^	^107^ SFPAFGTTGDAAT**R**	33
	Beta Tubulin 5	Eucgr.D01847.1	148^1^	^155^ IREEYPDR	50
	Elongation factor 1-alpha	Eucgr.B02473.1	498^1^	^165^ ARYDEIVK	49
	Heat shock protein 70 kDa	Eucgr.J00023.1	382^1^, 204.21^2^	^38^ NRTTPSYVAFTDTER ^478^ DNNLLGKFELSGIPPAPR, ^540^ TTPSYVGFTDTER	69

List of proteins identified from bands of whole cell lysates that correspond to methylated banding as determined by Western blotting. Score determined by either Mascot (1) or Peaks (−10logP) (2). Methylated arginines within sequences indicated in bold (dimethylated) or underlined (monomethylated)

Methylation of β-tubulin by EgPRMT1 was demonstrated using immunoprecipitation and Western blotting. Protein extracts from control and *35S::EgPRMT1* roots were immuno-precipitated with a β-tubulin antibody and the resulting protein was visualized using Western blots with both the ASYM24 and β-tubulin antibodies (Fig. 7b). The relative amount of methylated β-tubulin is, on average, 14x higher in the *35S::EgPRMT1* root sample than in the control (expressed as a fold-change of methylated tubulin over total β-tubulin calculated from Western blot band intensities).

## Discussion

We have demonstrated an important role for Type I PRMT homologues in the development of root tissues in *E. grandis*, an economically important model tree species. Unlike the better studied *A. thaliana*, *E. grandis* encodes only one PRMT4 homologue and one PRMT1 homologue, giving it the smallest complement of PRMT homologues out of the plant species considered. All seven of the identified PRMT homologues in *E. grandis* are expressed in a similar pattern in the various plant tissues investigated, even though methylated proteins vary by tissue. This suggests that despite similar gene expression profiles, PRMT substrates may vary between tissues and/or that PRMT proteins are subject to a level of control beyond transcription. The activity of PRMTs can be modified through phosphorylation [4, 35, 36], regulation by PRMT-binding proteins [37, 38] or by automethylation [39, 40]. Through mass spectrometry a broad selection of proteins from both root and leaf tissue were identified as having methylated arginine residues. Of these proteins, a large number are enzymes involved in photosynthesis or cellular respiration. Many of these have been previously described as having methylated arginine residues in other systems [41, 42]. Other proteins identified (e.g. Hsp70, 14-3-3, tubulin) have been associated with roles in maintenance of root architecture [30–32, 43, 44]. Previous work in tomato roots identified Hsp70 as methylated [45], and α- and β- tubulin were reported to be methylated in mammalian tissues [46], however methylation of 14-3-3 like proteins has not been previously described. It is likely that there are many more peptides and proteins methylated in *E. grandis* that were not detected in this study, but our results demonstrate that PRMTs of *E. grandis* appear to target regulatory proteins in a tissue-specific manner. Overall, the overlap between discovered methylated proteins in our system and those previously described indicate that there is some consistency and conservation in the targets of PRMTs, regardless of organism.

Chemical inhibitors of PRMT activity caused a reduction in growth when applied to *E. grandis* roots. Microscopic observation of the roots showed that the lack of growth in the roots may be due to a disturbance in root tip maintenance as both the meristem and elongation zones were significantly shortened. The results of the inhibitor experiments were complemented with transgenic mis-expression of Type I PRMTs in *E. grandis* roots, with RNAi silenced roots having both reduced growth and fewer lateral roots. Previous studies in fungal model systems have demonstrated a similar effect of PRMT activity on cellular extension and tissue growth, with deletions of *PRMT1* or *PRMT3* analogues resulting in significantly reduced hyphal elongation or hyphal branching, respectively [47, 48]. The transcriptomic analysis presented here of roots treated with DMNS – inhibiting only PRMT1 activity - showed a large number of significantly regulated genes, 128 of which have proposed roles in root development, including meristem maintenance according to PFAM enrichment analysis. A large number of enzymes involved in the formation of cell walls (eg. laccases, xyloglucan endotransglucosylases or cellulose synthases) are differentially regulated in tissues inhibited in PRMT1 activity. This may play a role in the lack of growth seen in treated and transgenic roots. Additionally, many regulated proteins are protein kinases or transcriptional regulators. Therefore, we find that PRMTs not only methylate and change the function of important signalling molecules directly (as seen with the protein data) but that they also affect the transcription of other regulatory molecules. Thus PRMTs are likely to be positioned near the top of a signalling cascade as major regulators of cellular processes.

We found that the transgenic mis-expression of four different Type I PRMT genes elicited similar root phenotypes. Additionally, recent work has shown that repression of PRMT5 (Type II PRMT) in *Arabidopsis* also results in shorter roots [17, 22]. This is an argument both for and against redundancy within the PRMT family as all of these PRMTs seem to accomplish a similar end result and yet are unable to “make up” for the loss of one another. A recent study by Hernandez et al. [49] demonstrated that the morphological and transcriptomic phenotype of *A. thaliana* to the loss of either *AtPRMT4* or *AtPRMT5* also had significant overlaps. One reason for this could be the tendency of PRMTs to form homo- or hetero-dimers with each other, thus multiple PRMTs may be necessary for the same methylation event [50]. Alternatively, the proper function of a protein or signalling pathway may require methylation at multiple arginine residues by separate PRMTs and thus the loss of any one PRMT is deleterious. Finally, it is probable that PRMTs have such a crucial role in plant processes that the loss of any one of them severely compromises the health and vitality of the plant root causing the observed short root phenotype, though this may occur via a different mechanism for each PRMT homologue.

In mammalian systems, PRMT1 is the most active arginine methyltransferase, accounting for up to 80% of asymmetric arginine methylation [8], and preferentially methylates arginine residues within Glycine-Arginine rich (GAR) motifs [51]. Within young *E. grandis* seedlings, *EgPRMT1* is not the most highly expressed *PRMT* gene although Western blot analysis of *35S::EgPRMT1* root proteins shows a greater increase in methylated proteins as compared to *35S::EgPRMT10* roots. This latter result could be due to a bias of the ASYM24 antibody generated against the GAR motif preferentially methylated by PRMT1 [52]. Arguing against this bias, however, was the finding that the majority of the peptides with methylated arginine residues identified by mass spectrometry did not encode a GAR motif: only 12% of the methylated arginine residues identified had a neighbouring glycine residue. Therefore, while it is possible that PRMT1 plays a predominant role in asymmetric methylation of *E. grandis* proteins as has been observed in mammalian systems, it is likely that it methylates more than just GAR motifs.

Overexpression of *EgPRMT1* results in a root hair phenotype not seen with the other *E. grandis* PRMTs studied. Normal root hairs grow only from the tip in a finely balanced cooperation between microtubules and the actin cytoskeleton [53]. Tubulin has been found to control root morphology in *Arabidopsis*, where reduced levels of tubulin correlated with aberrant microtubule assembly, laterally expanded root width, reduced root growth and altered root hair density and morphology [34]. Both actin and tubulin proteins have been identified as containing methylated arginine residues in mammalian systems [25, 46, 54]. Our own analysis identified α- and β-tubulin as methylated in *35S::EgPRMT1* tissues. Further, immunoprecipatation and Western blot analysis demonstrated a 14 times increase in the relative amounts of methylated β-tubulin within *35S::EgPRMT1* tissues, identifying β-tubulin as a substrate of EgPRMT1. As increased methylation has been proposed to cause microtubule destabilisation in mammalian tissues [25], it is reasonable to suggest that the over-methylation of β-tubulin in EgPRMT1 overexpressing plants could be one of the factors contributing to the abnormal root hair phenotype. Additionally, xyloglucan endotransglycosylase activity is necessary for root hair initiation in *Arabidopsis* [55] and our transcriptomic data demonstrate that several xyloglucan endotransglycosylases are upregulated in inhibitor treated tissues, implicating EgPRMT1 as a repressor of their expression. Thus, the observation of rounded root hairs in *35S::EgPRMT1* could occur directly, as a result of excessive methylation of cytoskeletal proteins, and/or indirectly through alteration of cellular signalling.

## Conclusions

PRMTs are important, well-conserved proteins found in the genomes of all eukaryotic organisms described to date. *Eucalyptus grandis* encodes its own set of seven PRMT homologues that are expressed in all plant tissues and methylate a variety of proteins involved in photosynthesis, cellular respiration and signalling. Investigation into the role of Type I PRMTs in the development of *E. grandis* roots demonstrates a crucial role for these proteins in the growth and branching of plant roots and root hair initiation. Our results concerning the impact of EgPRMT1 gene expression on root hair morphology also demonstrate that, while PRMTs are crucial in many cellular processes, their over-production can also have negative effects. Therefore, these genes must be carefully regulated within cells. The mechanisms by which PRMTs alter root morphology, however, require further studies as well as an investigation into the roles of the individual plant proteins methylated by PRMTs and their downstream effects.

## Methods

### Construction of PRMT phylogeny in model plant species

PANTHER protein classification PTHR11006 and the PFAM conserved domain PF05185 were used to retrieve all PRMT containing sequences in the genomes of *E. grandis*, *A. thaliana*, *O. sativa*, *G. max*, *P. trichocarpa*, and *S. purpurea* from Phytozome v10.3 (phytozome.jgi.doe.gov: accessed 29/10/2015). A phylogenetic tree was constructed using the online tool ‘Phylogeny.fr’. All of the PRMT-like protein sequences were downloaded from the Phytozome database. PRMT homologue assignment was based on prior annotation of the *A. thaliana* genome (http://www.arabidopsis.org/).

### PRMT expression in plant tissues

*Eucalyptus grandis* (W. Hill ex Maiden) seeds were obtained from the Commonwealth Scientific and Industrial Research Organisation (CSIRO, Clayton, Vic., Australia) tree seed center (Seedlot 21068). They were sterilized in 30% H_2_O_2_ for 10 min, followed by several washes in sterile water. Seeds were germinated on 1% agar media and transferred after one month to MS media. A sterile cellophane membrane was placed on the surface of the MS media to prevent the roots from growing into it. Plants were grown on the MS media for another six weeks with a 16 h photoperiod and at a temperature of 25 °C and were then harvested. Three independent biological replicates of leaves, stem, shoot apex and roots were harvested separately and immediately frozen in liquid nitrogen. RNA was extracted from the tissues using the Qiagen RNeasy Plant Mini kit as per manufacturer’s instructions. RNA was made into cDNA with the iScript cDNA synthesis kit (Bio-Rad). The SensiFAST SYBR No-ROX kit (Bioline) was used for qPCR analysis on a Corbett Rotor-gene 6000 RT-PCR cycler. Relative expression levels were calculated as the difference in expression as compared to the control genes, Eucgr.C00350.2 and Eucgr.K02046.1. Primer sequences used can be found in Additional file 4: Table S4. Expression values of PRMTs in mature *Eucalyptus* tissues was retrieved from https://phytozome.jgi.doe.gov/pz/portal.html#!info?alias=Org_Egrandis (accessed November 2016).

### Protein extraction and mass spectrometric identification of methylated proteins in plant tissues

A mass of 250 mg of fresh *E. grandis* leaf or root tissues was harvested and snap frozen in liquid nitrogen. Tissues were then ground immediately after freezing in a sintered glass tissue grinder in either ice cold 1x IP Lysis/Wash Buffer (Thermo Scientific) supplemented with 1 mM plant protease inhibitor cocktail (Sigma Aldrich; Cat#P9599) for immunoprecipitation or in 50 mM Tris HCl (pH 8)/1% SDS/50 μM DTT/1% PVPP/1 mM plant protease inhibitor cocktail for total protein extraction and Western blotting. Grinding was performed on ice for no more than 3 min after which the soluble protein was quantified using the Qubit total protein analysis kit according to manufacturer’s instructions (Life Technologies). Protein extract was then diluted to 1 μg/μL using the extraction buffer and either used immediately for immunoprecipitation or was mixed with NuPAGE LDS buffer (Life Technologies) and snap frozen in liquid nitrogen for Western blotting.

Immunoprecipitation was performed using the Pierce IP immunoprecipitation kit (Thermo-Scientific; Cat#26148) where ASYM24 (Merck-Millipore; Cat#07-414) or anti-β-tubulin (Abcam; Cat#ab6046) was cross-linked to the agarose resin. Approximately 900 μg of total protein was added to the agarose slurry and incubated at room temperature for 2 h. Following incubation, rinses and elution of the bound proteins were performed according to manufacturer’s instructions.

For protein separation by electrophoresis, equal amounts of total protein were separated on a 4–20% Mini-PROTEAN® TGX™ gradient gel for 2 h at 80 V. The gel was stained overnight, either with Brilliant Blue G- Colloidal stain or SYPRO Ruby, at room temperature with shaking. Coomassie stained gels were destained in milli-Q water for 5 h. Protein bands were excised and further destained for 10 min in a 1:1 solution of 25 mM ammonium bicarbonate and acetonitrile. This was repeated until bands appeared colourless, and was followed by incubation with acetonitrile for 20 min. Bands in SYPRO Ruby stained gels were visualised by UV Transilluminator, bands were excised and then destained as per the Coomassie stained bands. All further treatments were identical for gel bands stained with either staining method.

The gel bands were incubated with 10 mM DTT in milli-Q water for 1 h at 37 °C to reduce cysteine residues, then with 25 mM IAA in milli-Q water for 1 h at 37 °C to alkylate the cysteine residues. Proteins were digested by the addition of Trypsin Gold (Promega) with a final enzyme concentration of 5 μg/mL and incubated overnight at 37 °C. While trypsin was used to cleave proteins, it has been reported to produce inconsistent [46] or ineffective cleavage at di-methylated arginine sites [56]. The digestion solution was collected into new low-binding tubes and peptides were further extracted by adding a 1:1 solution of acetonitrile and 0.1% formic acid and sonicated for 10 min. This process was repeated twice. Peptides were dried under vacuum (Waters) and were then resuspended in 0.1% formic acid.

LC-MS/MS analysis was performed on a Xevo QToF mass spectrometer from Waters (Micromass,UK) fed by a nanoAquity UPLC (Waters Corp., Milford, MA, USA) at the Western Sydney University mass spectrometry facility. 3 μl of digested peptides were loaded onto a nanoAquity UPLC Symmetry C18 trapping column (1.7 μm, 180 μm × 20 mm) and then separated and eluted from the column using a binary gradient program at a flow rate of 5 μl/min and desalted at this flow rate for 3 min. The peptides were washed off the trap at 400 nL/min on to a Waters C18 BEH analytical column (75 μm x 100 mm), packed with 1.7 μm particles with 130 Å pore size. After separation, the peptides were analysed using tandem mass spectrometry, implementing an emitter tip that tapers to 10 μm at 2300 V. Mobile phase A was 0.1% formic acid in water; and mobile phase B was 0.1% formic acid in acetonitrile. The nano-UPLC gradient was as follows: 0 min, 97:3 A/B; 5 min, 97:3 A/B; 75 min, 40:60 A/B; 85 min, 10:90 A/B; 97 min, 97:3 A/B; 110 min, 97:3 A/B. The mass spectrometer was operated in positive ESI mode with capillary voltage of 3.5 KV, cone voltage of 40 V, source temperature of 80 °C. Targeted MS/MS data or DDA (data dependent acquisition) data were acquired by continuously scanning for peptides of charge state 2^+^ to 4^+^ with an intensity of more than 50 counts per second; with a maximum of three ions in any given 3 s scan. Selected peptides were fragmented and the product ion fragment masses were measured. The data were acquired by the software Masslynx (Version 4.1, Micromass, UK).

The acquired DDA data from Masslynx with “RAW” extension, were converted to PKL files by Proteinlynx Global Server (PLGS) for analysis using the Mascot Daemon database (Australian Proteomics Computational Facility, Melbourne, Australia).

The MS/MS data files were searched against MSPnr100 database with trypsin as the enzyme. The following parameters were considered in the Mascot Editor tab for identification of the peptides: maximum missed cleavage of 3, peptide charge state of 2^+^ and 3^+^, peptide mass tolerance of 0.5 Da in MS and MS/MS data base, fixed modification: carbamidomethyl (C) and variable modifications: oxidation (M), mono and di-methylation of arginine (R). An ion score of 20 was applied to all results in order to filter out low probability matches. Peptides were also matched using Peaks Studio Software (version 7.5; Bioinformatics Solutions Inc., Waterloo, Ontario, Canada) by uploading the *E. grandis* protein list translated from the primary transcripts [24] and performing a database search to identify proteins and post-translational modifications. The following parameter settings were used: fixed modifications: carbamidomethyl (C); variable modifications: oxidation (M), mono and di-methylation of arginine (R); enzyme: trypsin; number of allowed missed cleavages: 3; peptide mass tolerance; 100 ppm; MS/MS mass tolerance: 0.5 Da; and peptide charge state: 2^+^ and 3^+^. Proteins identified as uncharacterised proteins were matched to their function by performing a BLAST search (Uniprot) and homology of the identified residues was checked using the alignment tool (Uniprot).

A recent report by Hart-Smith et al. [57] warned of false positive detection of methylated peptides when using ethanol, methanol and/or isopropanol in the preparation of large-scale mass spectrometry. The methods used here were different to the ones described by Hart-Smith [57] and the results were validated in the absence of alcohol-based staining. Furthermore, bacterially expressed tubulin β (which lacks methylation, Sigma; Cat#SRP5148) analysed from stained or unstained protocols, did not yield any falsely identified methylated arginine residues using the mass spectrometry protocol described above.

### Western blot analysis

For Western blot analysis, equal amounts of protein were separated on a 4–20% Mini-PROTEAN® TGX™ gel for 2 h at 80 V. Proteins were then transferred onto a Nitrocellulose membrane (Bio Rad) for 1 h at a constant voltage of 35 V using an X-Cell II Blot Module in an Invitrogen Novex™ Mini-Cell. The buffer used was *NuPAGE* transfer buffer (Cat# NP0006). The membranes were blocked for 1 h in 3% skim milk powder dissolved in Tris-buffered saline (TBS; 50 mM Tris-Cl/150 mM NaCl pH 8.0). After blocking, the membrane was washed with TBS 3x followed by overnight incubation at 4 °C in the primary antibody diluted in TBS as follows: 1:500 dilution of ASYM24 (for detection of asymmetrically methylated proteins) or 1:500 dilution of anti-tubulin (as a loading control protein). The membranes were then washed 3x in TBS and then incubated for 2 h at room temperature in an HRP-conjugated anti-rabbit antibody (Merck-Millipore; Cat#12-348). Following another 3x washes in TBS, the membranes were treated with Clarity™ ECL substrate for 5 min and the chemiluminescent signal was captured using a VersaDoc Imaging System (Bio Rad). All Western blots presented in the main text are representative images of multiple blots. Each sample type was extracted in a minimum of biological triplicates with the exception of transgenic tissues, which were each treated as a single transformed line.

### Inhibitor trials – roots only

Young *E. grandis* seedlings were grown as above – germinating for one month on 1% agar and then growing for an additional six weeks on MS media covered with a sterile cellophane membrane. Their roots were cut and allowed to commence regrowing to ensure an active growth phase. At T_0_, the starting length and position of the roots were recorded. Roots were treated with either AdOx (5 nM-5 μM), AMI-1 (50 nM-50 μM), 2,3-dimethoxynitrostyrene (30 nM-30 μM in 1% DMSO), 1% DMSO control or water control and root growth was measured 24 h later. The first inhibitor, AdOx, is a general methylation inhibitor and indirectly blocks the reaction providing the methyl group that is added by methyltransferases to their respective substrates including arginine. Thus all methylation processes, including lysine methylation, are inhibited by AdOx. The second inhibitor, AMI-1, more specifically inhibits protein arginine methylation by specifically blocking PRMTs but not PKMTs [58]. The more recently described DMNS only inhibits the activity of PRMT1 (and PRMT8, not present in *E. grandis*; [59]). Alignment of PRMT protein sequences from human and *E. grandis* genomes shows that *EgPRMT1* has the appropriate cysteine residue to be inhibited by DMNS, while the other *E. grandis* PRMTs do not (Additional file 5: Figure S1). One millilitre of solution was administered directly to each root system daily over the test period of two weeks. Between 17 and 30 replicates were performed for each treatment type. Lateral rooting was analyzed after two weeks of treatment. Root tip samples were taken and observed with a Zeiss Stemi 2000-C stereomicroscope (Germany). All plants were alive and healthy at the time of harvest, indicating that the dosage of inhibitors was sub-lethal.

### Transcriptomic analysis of DMNS treated roots

Three representative biological replicates of roots from DMNS treated roots and 1% DMSO treated control roots were snap frozen in liquid nitrogen and the RNA was extracted using the Qiagen RNeasy Plant Mini kit as per manufacturer’s instructions. Transcriptional analyses of all tissues were performed using RNA-Seq via conventional poly-A library preparation for Illumina sequencing (TruSeq RNA Library Prep v2, Illumina). Library construction and 100-bp paired-end reads sequencing was performed by the Western Sydney University Next Generation Sequencing Facility on three independent biological replicates of DMSO-control treated roots and DMNS treated roots. The samples were indexed and run on four high-output lanes (paired end, 1.4 billion perfect reads) of an Illumina Hi-Seq 2000 flow-cell. Raw reads were trimmed for quality and aligned to the primary transcripts of the *E. grandis* genome taken from https://phytozome.jgi.doe.gov/pz/portal.html#!info?alias=Org_Egrandis (accessed November 2015; [24]) using CLC Genomics Workbench 7. For mapping, the minimum length fraction was 0.9, the minimum similarity fraction 0.8, and the maximum number of hits for a read was set to 10. The unique and total mapped reads number for each transcript were determined, and then normalized to reads per kilobase of exon model per million mapped reads. CLC software was used to determine the statistical significance of gene expression. To identify functional domains that were significantly enriched, PFAM annotation of the differentially regulated genes was analyzed by the dcGO program http://supfam.org/SUPERFAMILY/cgi-bin/dcenrichment.cgi (accessed December 2015). Only FDR-corrected of PFAM slim ‘specific’ and ‘highly specific’ annotations with *p*-values < 0.001 were assigned as significantly over-represented in the data set (Additional file 2: Table S2 and Additional file 6: Table S3).

### Production of PRMT *35S::*overexpression and RNAi transgenic roots

*EgPRMT1*, *EgPRMT10*, *EgPRMT3* and *EgPRMT4* were cloned from cDNA synthesized using iScript (Bio Rad) from total RNA extracted from *E. grandis* roots using RNeasy plant extraction kit according to manufacturer’s instructions (Qiagen). The amplified fragments were gel purified and ligated into pDONR222 and sequence verified. Positive inserts were then transferred to pH2GW7 (*35S::*) or to pH7GWIWG2(II) (RNAi) using Gateway Gene Cloning (Life Technologies) and transformed into *Agrobacterium rhizogenes* isolate K599. *E. grandis* seedlings were grown from seed to one month old on 1% agar media. The root system of the plant was cut off and the resultant wound was dipped in freshly grown *A. rhizogenes* expressing the plasmid of choice, or wild type control. Dipped plants were embedded in MS media and left for one week, placed upside down, in a growth cabinet with a constant temperature of 25 °C and a 16 h photoperiod. Plants were then transferred to fresh MS media supplemented with 150 μg/mL Timentin and grown under the same conditions [60]. Transformed roots typically emerged within one or two weeks. After one months of growth, the transgenic roots were harvested. Harvested roots were scanned and measured (using ImageJ), analysed by microscopy (Zeiss Stemi 2000-C, Germany) and frozen at −80 °C. RNA was extracted from select transformed roots, made into cDNA and analyzed by qPCR to confirm altered expression of the transgene. Growth data for each individual transformed line is given in Additional file 3: Figure S2. Antibodies that cross-react with *E. grandis* PRMT proteins are only commercially available for *EgPRMT1.* Therefore, we were only able to confirm increased abundance of the EgPRMT1 protein in the transgenic roots (Additional file 7: Figure S3). RNAi lines were not similarly probed as total protein recovered was not sufficient to detect EgPRMT1 signal.

## Additional files

Additional file 1: Table S1. Listing of genes exhibiting significant differential expression levels in roots treated with DMNS as opposed to control treated roots. Genes were considered to be significantly regulated when fold change in gene expression was ≥ 5-fold, *p* ≤ 0.05. (XLSX 105 kb)
Additional file 2: Table S2. Significantly enriched PFAM classifications in genes with elevated expression levels in roots treated with DMNS as opposed to control treated roots. (XLSX 51 kb)
Additional file 3: Figure S2.
*Agrobacterium rhizogenes* transformed *E. grandis* roots overexpressing (*35S::*) or with RNAi silenced (RNAi) version of selected Type I PRMT homologues – *EgPRMT1*, *EgPRMT10*, *EgPRMT3* and *EgPRMT4..*(A) Primary root length of each independent transgenic line; (B) Lateral root density of each independent transgenic line; (C) Root meristem size for each independent transgenic line; (D) Size of the root elongation zone for each independent transgenic line. (JPG 95 kb)
Additional file 4: Table S4. Quantitative PCR and gene cloning primers used in this study. (XLSX 10 kb)
Additional file 5: Figure S1. Alignment of PRMT amino acid sequence to demonstrate that the region upon which the inhibitor DMNS acts (bold, red cysteine residue) is only found in EgPRMT1. (JPG 58 kb)
Additional file 6: Table S3. Significantly enriched PFAM classifications in genes with reduced expression levels in roots treated with DMNS as opposed to control treated roots. (XLSX 48 kb)
Additional file 7: Figure S3. Western blot of EgPRMT1 in control *E. grandis* roots and in roots over-expressing *EgPRMT10* and *EgPRMT1.* Western blots of whole cell lysates from the roots of un-transformed, un-treated *E. grandis* roots, *E. grandis* roots transformed with *A. rhizogene* strain K599 (control) or roots over-expressing *EgPRMT1* or *EgPRMT10.* Blots were probed with anti-PRMT1. Equal amounts of total proteins were migrated in each sample and β-tubulin was used as a loading control. (JPG 34 kb)

## Abbreviation

aDMA: Asymmetric dimethyl arginine
AdOX: Adenosine dialdehyde
AMI-1: Arginine methyltransferase inhibitor 1
cDNA: Copy deoxyribonucleic acid
DDA: Data dependent analysis
DMNS: 2,3-dimethoxynitrostyrene
DMSO: Dimethyl sulfoxide
DNA: Deoxyribonucleic acid
DTT: Dithiothreitol
ESI: Electrospray ionization
GAR: Glycine Arginine rich
IAA: Indole-3-acetic acid
IP: Immunoprecipitation
LC-MS/MS: Liquid chromatography tandem mass spectrometry
LDS: Lithium dodecyl sulphate
MMA: Monomethyl arginine
PAGE: Polyacrylamine gel electrophoresis
PFAM: Protein family
PKMT: Protein lysine methyltransferase
PRMT: Protein arginine methyltransferase
PVPP: Polyvinylpolypyrrolidone
qPCR: Quantitative polymerase chain reaction
RNA: Ribonucleic acid
RNAi: Ribonucleic acid interference
SAM: S-Adenosyl methionine
sDMA: Symmetric dimethyl arginine
SDS: Sodium dodecyl sulphate
TBS: Tris-buffered saline
UPLC: Ultra performance liquid chromatography
