# Supplementary figures and images for: Root morphogenic pathways in Eucalyptus grandis are modified by the activity of protein arginine methyltransferases
Source: BMC Plant Biol. 2017 Mar 9;17:62. doi: 10.1186/s12870-017-1010-x (PMC5345158; doi:10.1186/s12870-017-1010-x)

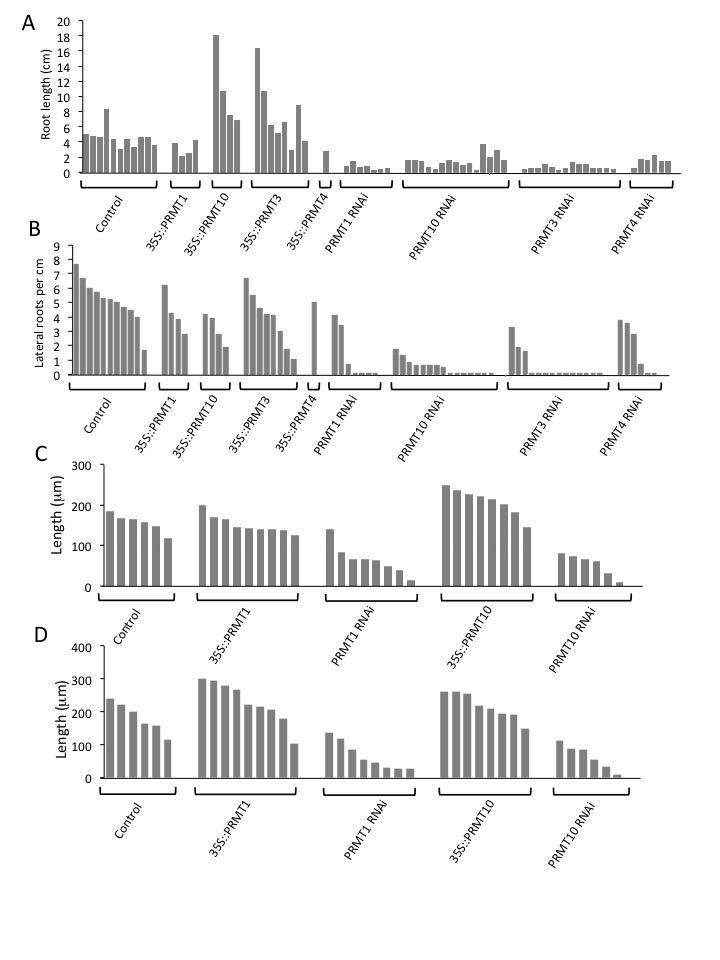

Supplement: Additional file 3: Figure S2. — Agrobacterium rhizogenes transformed E. grandis roots overexpressing (35S::) or with RNAi silenced (RNAi) version of selected Type I PRMT homologues – EgPRMT1, EgPRMT10, EgPRMT3 and EgPRMT4..(A) Primary root length of each independent transgenic line; (B) Lateral root density of each independent transgenic line; (C) Root meristem size for each independent transgenic line; (D) Size of the root elongation zone for each independent transgenic line. (JPG 95 kb) [file 12870_2017_1010_MOESM3_ESM.jpg]

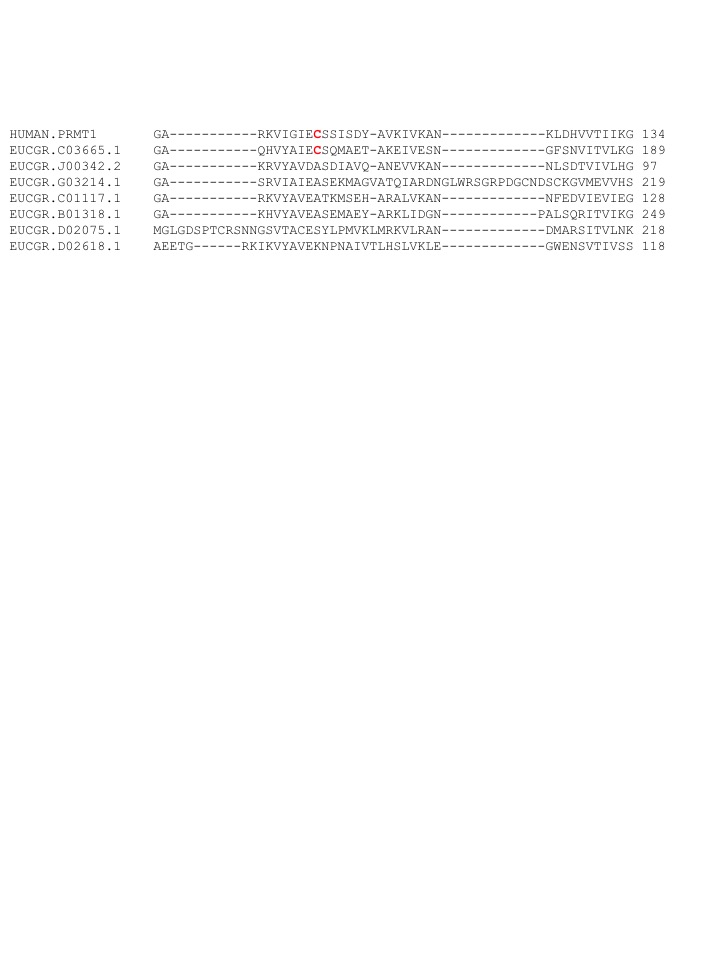

Supplement: Additional file 5: Figure S1. — Alignment of PRMT amino acid sequence to demonstrate that the region upon which the inhibitor DMNS acts (bold, red cysteine residue) is only found in EgPRMT1. (JPG 58 kb) [file 12870_2017_1010_MOESM5_ESM.jpg]

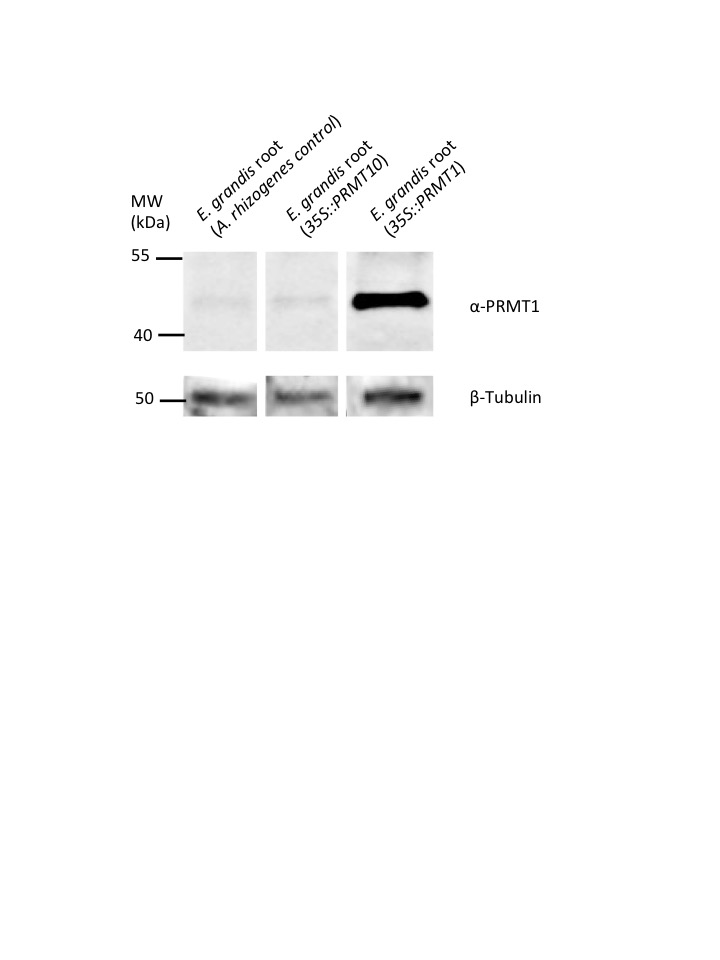

Supplement: Additional file 7: Figure S3. — Western blot of EgPRMT1 in control E. grandis roots and in roots over-expressing EgPRMT10 and EgPRMT1. Western blots of whole cell lysates from the roots of un-transformed, un-treated E. grandis roots, E. grandis roots transformed with A. rhizogene strain K599 (control) or roots over-expressing EgPRMT1 or EgPRMT10. Blots were probed with anti-PRMT1. Equal amounts of total proteins were migrated in each sample and β-tubulin was used as a loading control. (JPG 34 kb) [file 12870_2017_1010_MOESM7_ESM.jpg]
